# The clinical approach to small fibre neuropathy and painful channelopathy

**DOI:** 10.1136/practneurol-2013-000758

**Published:** 2014-04-28

**Authors:** Andreas C Themistocleous, Juan D Ramirez, Jordi Serra, David L H Bennett

**Affiliations:** 1 Nuffield Department of Clinical Neurosciences, John Radcliffe Hospital, Oxford, Oxfordshire, UK; 2 Neuroscience Technologies, Parc Científic de Barcelona, Barcelona, Spain; 3 Department of Neurology, MC Mutual, Barcelona, Spain

**Keywords:** NEUROPATHY, PAIN

## Abstract

Small fibre neuropathy (SFN) is characterised by structural injury selectively affecting small diameter sensory and/or autonomic axons. The clinical presentation is dominated by pain. SFN complicates a number of common diseases such as diabetes mellitus and is likely to be increasingly encountered. The diagnosis of SFN is demanding as clinical features can be vague and nerve conduction studies normal. New diagnostic techniques, in particular measurement of intraepidermal nerve fibre density, have significantly improved the diagnostic efficiency of SFN. Management is focused on the treatment of the underlying cause and analgesia, as there is no neuroprotective therapy. A recent and significant advance is the finding that a proportion of cases labelled as idiopathic SFN are in fact associated with gain of function mutations of the voltage-gated sodium channels Na_v_1.7 and Na_v_1.8 (encoded by the genes *SCN9A* and *SCN10A*, respectively). There is a further group of heritable painful conditions in which gain of function mutations in ion channels alter excitability of sensory neurones but do not cause frank axon degeneration; these include mutations in Na_v_1.7 (causing erythromelalgia and paroxysmal extreme pain disorder) and TRPA1 (resulting in familial episodic pain disorder). These conditions are exceptionally rare but have provided great insight into the nociceptive system as well as yielding potential analgesic drug targets. In patients with no pre-existing risk factor, the investigation of an underlying cause of SFN should be systematic and appropriate for the patient population. In this review, we focus on how to incorporate recent developments in the diagnosis and pathophysiology of SFN into clinical practice.

## Introduction

**Figure 1:**
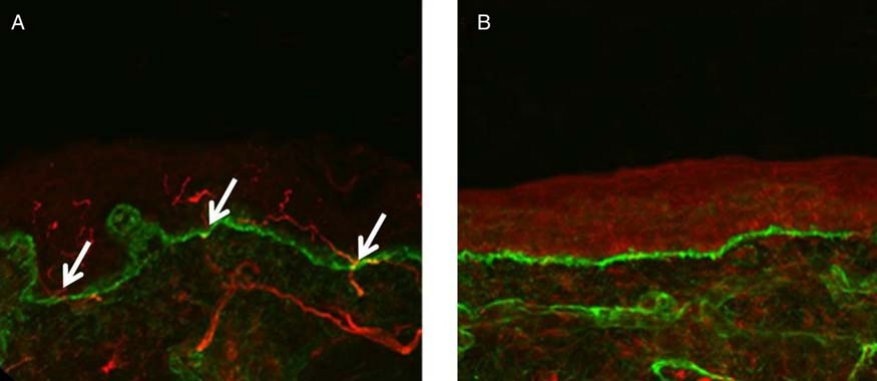
Confocal images of skin biopsies taken from the legs of a control subject (A) and a patient with small fibre neuropathy secondary to HIV (B) showing PGP 9.5-immunoreactive fibres (red) and the basement membrane (labelled with type IV collagen fibres, green). Nerve fibres positive for PGP 9.5 (white arrows) are counted as they cross the dermal–epidermal junction. The intra-epidermal nerve fibres are absent in the patient with HIV (B) consistent with the diagnosis of small fibre neuropathy. Scale bar: 50 µm.

Small fibre neuropathy (SFN) is defined as a structural abnormality of small fibres characterised pathologically by degeneration of the distal terminations of small fibre nerve endings^1^ ^2^ (figure 1). SFN complicates several common diseases, such as diabetes mellitus and HIV, and the associated pain contributes significantly to the morbidity of these diseases. Gain of function mutations in voltage-gated ion channels have recently been shown to cause SFN^3^ ^4^ and in addition can cause a number of heritable pain conditions in which small fibres are hyperexcitable yet remain structurally intact. These disorders are exceptionally rare but have provided great insight into the nociceptive system, in some cases revealing important targets for drug discovery. The clinical neurologist is likely to encounter SFN increasingly, given the rising prevalence of diabetes and improvements in the diagnosis of SFN.^5^ Furthermore, there is now greater clarity of diagnostic criteria. In this review, we will provide a framework for diagnosing and managing these conditions.

## What are the small fibres?

**Figure 2:**
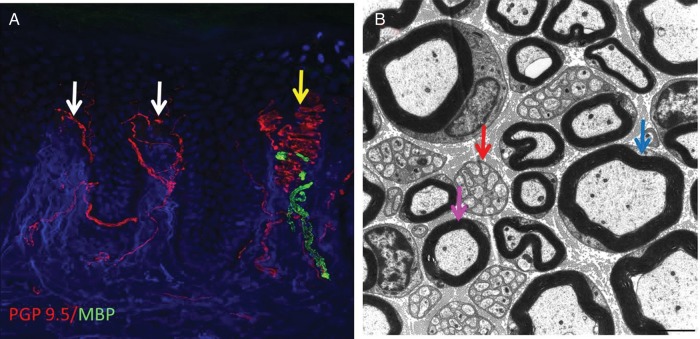
(A) Confocal image of a skin biopsy taken from the finger of a healthy subject illustrating different subtypes of sensory fibre: PGP 9.5 is used as an axonal marker (red) and myelin basic protein as a marker for myelin (green). There are numerous free nerve endings entering the epidermis (white arrows) and a single myelinated fibre is innervating a Meissner's corpuscle (yellow arrow). (B) Electron micrograph of the sural nerve. The blue arrow shows a large diameter myelinated fibre, the purple arrow marks a small diameter myelinated fibre and the red arrow marks a Remak bundle in which one non-myelinating Schwann cell associates with multiple unmyelinated C-fibres which are ensheathed in Schwann cell pockets.

Small fibres are small narrow diameter myelinated (Aδ) and unmyelinated (C) nerve fibres of the peripheral nervous system^6^ (figure 2). Somatosensory Aδ-fibres and C-fibres innervating skin pass through the dermis where they innervate cutaneous structures; both groups of fibres end as free nerve endings in the epidermis (the Aδ-fibres lose their myelin sheath as they cross the dermo-epidermal junction). Aδ-fibres are responsible for conveying cold input and nociceptive input. C-fibres convey innocuous warm sensations and possibly innocuous cold sensations,^7^ and noxious input from a variety of high threshold mechanical, thermal and chemical stimuli. Small fibres play an important role in the autonomic nervous system, because thin myelinated fibres contribute to preganglionic fibres and C-fibres contribute to postganglionic fibres,^8^ innervating structures such as sweat glands, blood vessels and the heart. Nerve growth factor (discovered in a series of groundbreaking experiments by the Nobel laureate Rita Levi-Montalcini^9^) is a key target-derived factor for these neurones. Mutations in nerve growth factor or its high-affinity receptor NTRK1 result in degeneration of nociceptors and sympathetic neurones; this leads to hereditary sensory and autonomic neuropathy type 5 and 4 respectively.^10^ The severe disability caused by painless injuries illustrates the vital importance of the nociceptive system.

## Aetiology, epidemiology and pathogenesis

The exact incidence and prevalence of SFN is unknown.^1^ ^11^ ^12^ There have been no satisfactory studies to assess the epidemiology of SFN, because until recently, there was no generally accepted definition for SFN and no standardised classification. There are a few natural history studies, and the general consensus is that in most patients the disease does not progress or progresses very slowly.^5^ ^12^

There are many potential causes of SFN (table 1),^1^ ^12^ the commonest being diabetes mellitus, responsible for approximately a third of all cases of SFN.^5^ In diabetes mellitus, a complex interplay of metabolic factors, ischaemia and impaired recovery predispose peripheral neurones, glial cells and vascular endothelial cells to damage that ultimately leads to neuronal injury and peripheral neuropathy.13–15 Interestingly, in those patients with SFN whom a diagnosis is not immediately apparent, a significant number have impaired glucose tolerance both at time of presentation or at subsequent follow-up, usually after about 1 year.^5^ However, the diagnosis of impaired glucose tolerance may merely reflect the underlying high incidence of impaired glucose tolerance in the population studied rather than a causal link. HIV and the continued use of neurotoxic antiretrovirals are probably the major causes of SFN in sub-Saharan Africa.^16^ HIV causes peripheral axonal injury through immune activation that creates a toxic microenvironment for peripheral nerves.^17^ Anti-retrovirals, specifically the nucleoside reverse transcriptase inhibitors such as didanosine and stavudine, cause axonal injury through mitochondrial toxicity.^16^ SFN occurs in several autoimmune and inflammatory diseases (table 1). The exact pathophysiological mechanisms are unknown, and the most likely candidate mechanisms include autoantibodies targeted against neuronal proteins (the identity of which is not yet known), elevated pro-inflammatory cytokines in the skin and dermal vasculitis.^1^ Amyloid neuropathy (either hereditary or acquired) can present as a pure SFN and usually progresses to involve the large neuronal fibres and major organs, such as the kidneys and heart.^18^ ^19^

**Table 1:** Causes of small fibre neuropathy

	
Primary	Secondary
Idiopathic ▸ Idiopathic small fibre neuropathy ▸ Burning mouth syndrome	Metabolic ▸ Impaired glucose tolerance ▸ Diabetes mellitus ▸ Rapid glycaemic control ▸ Vitamin B12 deficiency ▸ Dyslipidaemia ▸ Hypothyroidism ▸ Chronic kidney disease
Hereditary/genetic ▸ Na_v_1.7 mutations ▸ Na_v_1.8 mutations ▸ Familial amyloid polyneuropathy ▸ Fabry's disease ▸ Tangier's disease	Infections ▸ HIV ▸ Hepatitis C ▸ Influenza
	Toxins and drugs ▸ Anti-retrovirals ▸ Antibiotics—metronidazole, nitrofurantoin, linezolid ▸ Chemotherapy—bortezomib ▸ Flecainide ▸ Statin ▸ Alcohol ▸ Vitamin B6 toxicity
	Immune mediated ▸ Coeliac disease ▸ Sarcoidosis ▸ Sjögren's syndrome ▸ Rheumatoid arthritis ▸ Systemic lupus erythematosus ▸ Vasculitis ▸ Inflammatory bowel disease ▸ Paraneoplastic ▸ Monoclonal gammopathy/amyloid

Note that a number of these conditions may present as a small fibre neuropathy and then evolve to include large fibres.

SFN features in a number of rare genetic conditions (table 1). A recent study described variants of the *SCN9A* gene, which encodes the Na_v_1.7 sodium channel in a third of patients labelled as having idiopathic SFN.^20^ This voltage-gated ion channel is selectively expressed in sensory and autonomic neurones.^21^ Na_v_1.7 variants associated with SFN cause enhanced excitability of sensory neurones and eventual degeneration of small fibres, which is probably mediated through increased sodium load and reversal of sodium–calcium exchange.^22^ Na_v_1.8 is a related voltage-gated sodium channel selectively expressed in nociceptors. Variants in the *SCN10A* gene, which encodes the Na_v_1.8 sodium channel, enhance the excitability of dorsal root ganglion cells^23^ and are also associated with SFN. The penetrance of these variants in voltage-gated sodium channels has not yet been fully elucidated and in some cases these variants may be important risk factors, rather than being fully penetrant in causing SFN. This currently makes genetic counselling complex. Idiopathic SFN has historically accounted for 23–94% of cases.^24^ In our experience, approximately 50% of cases of SFN are idiopathic. However, this is highly dependent on referral patterns, and the number of idiopathic cases will reduce as genetic causes are identified.

## Clinical presentation

The symptoms of SFN vary between patients both in their severity and in their progression.^1^ ^5^ Typically the sensory symptoms begin in the feet and progress proximally in a length-dependent manner, eventually involving the hands ie, glove and stocking pattern. Pain is virtually always the presenting symptom and can be extremely severe and debilitating. Pain is usually ongoing (ie, stimulus independent), although some patients complain of evoked pain, for example, patients cannot tolerate bed sheets touching their feet or wearing socks. The pain is most often described as burning or prickling and can have a pruritic component. Pain attacks triggered by increased temperature or exercise may indicate painful channelopathies (Na_v_ 1.7 mutations) and Fabry's disease. Because of the lack of obvious neurological signs, patients may have seen multiple health professionals and been given a number diagnoses, including ‘plantar fasciitis’ or ‘collapsed arches’, in some cases leading to unnecessary surgery (which exacerbates pain). On direct enquiry, patients may comment on altered temperature sensibility, for example being unable to sense the temperature of a bath with their feet. Occasionally, SFN follows a non-length-dependent pattern with a patchy loss of function involving focal areas such as the trunk or face, particularly in the situation of auto-immune and inflammatory causes. It is important to enquire regarding autonomic nervous system dysfunction, which may include postural hypotension, gastrointestinal or sexual dysfunction. Symptomatic postural hypotension should always prompt consideration of amyloid neuropathy.

On clinical examination, there may be trophic changes such as dry, cracked or shiny skin over the affected areas. Typically (and to reach strict diagnostic criteria for SFN) motor function, deep tendon reflexes and coordination are normal, as is large fibre sensory function, such as light touch, vibration sensation and proprioception. There is often a distal loss of pinprick or thermal sensation, and punctate hyperalgesia and brush evoked allodynia may be present but are rare. On systemic examination, a postural drop in blood pressure with a resting tachycardia suggests autonomic nervous system involvement. There may be clues to underlying aetiology, such as peri-umbilical and bathing trunk angiokeratoma in Fabry's disease, characteristic enlarged tonsils in Tangier's disease and lymphadenopathy, organomegaly and thickened nerves associated with amyloid.

## Diagnostic criteria for SFN

The diagnostic criteria of SFN in diabetes mellitus have recently been reviewed and a clinical expert panel has derived a set of criteria that heavily emphasise the clinical features of SFN with associated special investigations. The diagnostic criteria are as follows^25^:

- Possible—length-dependent symptoms and/or clinical signs (pinprick and thermal sensory loss and/or allodynia/hyperalgesia).
- Probable—length-dependent symptoms, clinical signs of small fibre damage and normal nerve conduction studies.
- Definite—length-dependent symptoms, clinical signs of small fibre damage, normal nerve conduction studies, and altered intra-epidermal nerve fibre density at the ankle and/or abnormal quantitative sensory testing of thermal thresholds at the foot.

Lauria *et al*^12^ strongly recommend using these criteria in SFN of any cause, irrespective of whether the neuropathy is length- or non-length dependent. At the moment, the best evidence base for the diagnosing SFN is the combination of clinical signs of small-fibre dysfunction and reduced intra-epidermal nerve fibre density (rather than altered thermal thresholds, which have a reduced diagnostic efficiency compared with clinical examination).

### Electrodiagnostic studies

**Figure 3:**
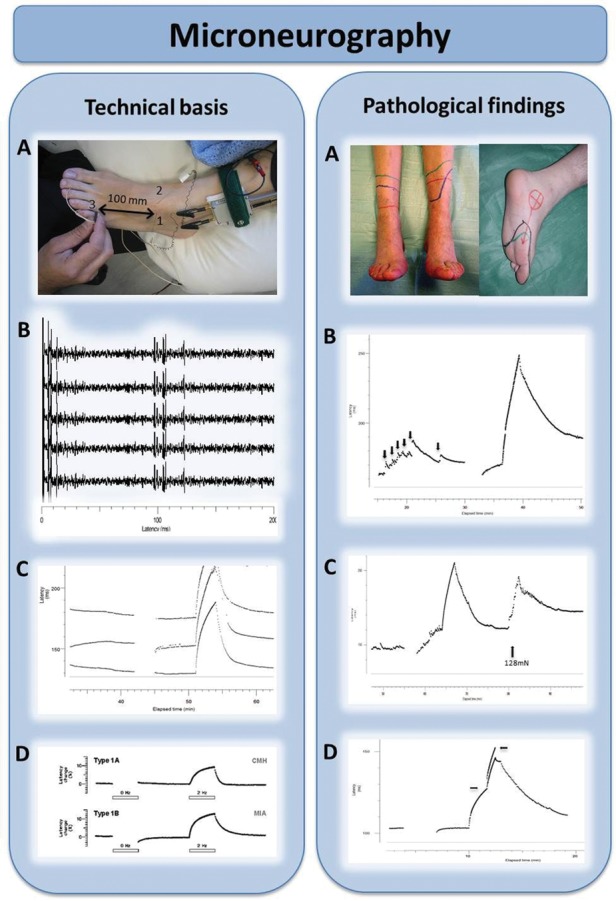
Microneurography in humans: microneurography is an electrophysiological technique to record action potentials from individual peripheral nerve axons in humans. Technical basis (left). **(A) Microneurography setting.** A tungsten microelectrode (1) is inserted intraneurally in a peripheral nerve; in this case the superﬁcial peroneal nerve at the dorsum of the foot. A subcutaneous reference electrode (2) is inserted in the skin outside the nerve trunk. The innervation territory of the nerve is electrically stimulated with a pair of needle electrodes (3). In this example, conduction distance between stimulating electrodes and an active microneurography electrode is 100 mm. **(B) Electrophysiological recording of responses.** Electrical stimulation of the receptive field evokes multiple action potentials in the sweep. Latencies of these action potentials allow segregation of units between a group of thinly myelinated, Aδ units (latencies ≈ 10 ms; conduction velocity ≈ 10 m/s) and a group of unmyelinated C-units (latencies ≈ 100 ms; conduction velocities ≈ 1 m/s). Superposition of the individual sweeps in a cascade mode demonstrates reproducibility of the responses. **(C) Raster plot of latencies.** The individual sweeps displayed in a cascade mode in B can also be displayed as a raster plot of latencies, with latency as the y-axes and elapsed time as the x-axes. In this example, three C-units at approximate latencies of 140, 155 and 180 ms at min 40 are displayed. Unmyelinated C axons change their conduction velocity (and hence their latency) depending on the stimulation frequency, a phenomenon called ‘activity-dependent slowing of conduction velocity’. For example, baseline stimulation at 0.25 Hz can be interrupted (open bar) or increased to 2 Hz (filled bar). This induces changes in latency with different profiles. **(D) Profiles of activity-dependent slowing of conduction velocity.** Different subpopulations of peripheral unmyelinated axons display different profiles of conduction slowing. For example, C-nociceptors have characteristic ‘shark fin’ profiles. **Pathological findings (right). (A) Neuropathic pain.** Peripheral nerve damage, such as an axonal polyneuropathy (left) or a posterior tibial nerve damage (right) frequently induce positive sensory phenomena. Burning or deep aching pain, due to activity arising from C-nociceptors, is a frequent complaint in neuropathic pain patients. **(B) Spontaneous discharges in C-nociceptors.** Damaged C-nociceptors frequently engage in ongoing, spontaneous activity. Spontaneous discharges are indicated by abrupt increases in latency (black arrows) between successive electrical stimuli delivered at 0.25 Hz, giving rise to a characteristic ‘saw-tooth’ profile of the raster plot. **(C) Peripheral sensitisation in C-nociceptors.** Example of mechanically evoked activity in a normally mechano-insensitive C-nociceptor recorded from a patient suffering from small-fibre neuropathy. Spontaneous discharges are evoked by pressing with a von Frey hair exerting a force of 128 mN (arrow). **(D) Double spike in pathological C-nociceptor.** Under normal conditions, single electrical shocks into the receptive field of C-nociceptors induce single action potentials. In neuropathy, with frequent dying-back and regeneration attempts, extensive, abnormal branchings may induce ‘unidirectional blocks’ in the terminal arborisation of the units, and induce multiple spikes (arrows signal beginning and end of the double spike), thus amplifying peripheral input. Double and triple spikes may contribute to hyperalgesia in patients with neuropathic pain.

The major limitation of conventional nerve conduction studies is that they primarily assess only large myelinated fibres and cannot detect any change in small fibres. In pure SFN, conventional nerve conduction studies will be normal and therefore their purpose is to exclude an associated large fibre component, with implications for the differential diagnosis. Lasers can be used to stimulate Aδ-fibres, and the associated evoked cortical potentials (laser-evoked potentials) allow interrogation of somatosensory pathways from the terminations of Aδ-fibres in the skin to the cortex.^26^ Microneurography (figure 3) uses recording microelectrodes placed within nerve fascicles and the ‘marking’ technique enables multiple small fibres to be recorded from simultaneously.^27^ This is a potent resource for research as the functionality of small fibres can be directly determined (figure 3) and with advancing technology is likely to be used more in clinical practice.

### Quantitative sensory testing

**Figure 4:**
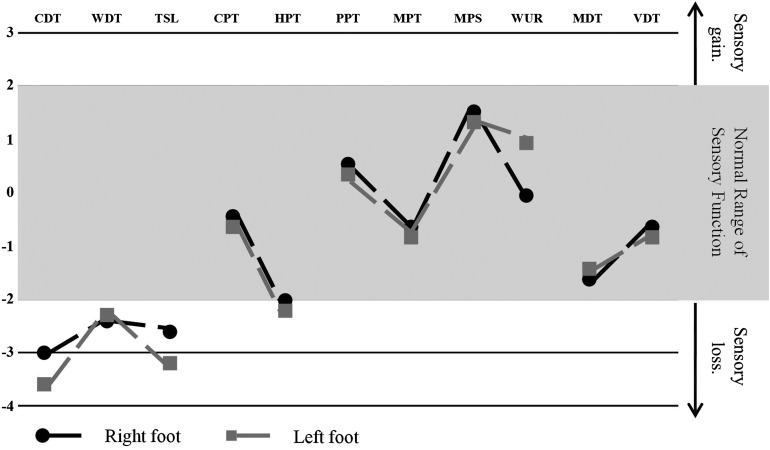
An example of a quantitative sensory testing profile for a patient with small fibre neuropathy. Data are reported as z-score profiles for each sensory test as depicted here. Z-score is defined as the SD of the recorded result from the mean normal data result and calculated as z=(value _patient_−mean _controls_)/SD_controls_. Each data point is discrete; however, they are connected for graphical illustration as a z profile. Quantitative sensory tests included CDT, cold detection threshold; WDT, warm detection threshold; TSL, thermal sensory limen; CPT, cold pain threshold; HPT, heat pain threshold; PPT, pressure pain threshold; MPT, mechanical pain threshold; MPS, mechanical pain sensitivity; WUR, wind up ratio; MDT, medical detection threshold; and VDT, vibration detection threshold. Normal data are distributed within the shaded area (mean at 0±2 SD). Note selective and significant impairment of small fibre function including hyposensitivity to cooling, warming, elevated heat pain threshold and impaired thermal sensory limen.

QST is a psychophysical investigative tool to assess the function of the human somatosensory nervous system.^28^ A variety of mechanical and thermal challenges, non-nociceptive and nociceptive, of measured intensity provide an assessment of the function of Aβ-fibres, Aδ-fibres and C-fibres and their corresponding central pathways. In the field of pain research, there are increasing efforts to link particular patterns of sensory dysfunction—detected through symptom questionnaires and QST—to underlying pathogenic mechanisms.^29^ Several variables, such as detection thresholds, pain thresholds and stimulus–response functions, can be measured. For example, in the German Research Network on Neuropathic Pain (DFNS) protocol, seven sensory tests generate 13 parameters of sensory function.^30^ One recent advance is the generation of large normative data sets of age-matched and sex-matched controls.^30^ QST has been adopted by a number of associations as part of peripheral neuropathy and neuropathic pain assessment.^31^ In assessing SFN, thermal detection and pain thresholds are commonly used (figure 4). However, several factors will affect the reproducibility of QST measurements, including the training of both examiner and patient, the methodology of assessment, baseline skin temperature, the stimulus characteristics, the location and number of stimulus sites and the duration of intervals between tests.^28^ There has been an increasing effort to standardise QST protocols and training in order to reduce variability.^30^ ^32^ It should be noted that QST cannot differentiate between peripheral and central causes of a sensory deficit, measurements will be confounded by poor concentration or a cognitive deficit, and cut-off values are required to avoid skin damage thus making extreme end points unreliable. In summary, QST is a powerful research tool especially when applied to populations but is inadequate as a stand-alone assessment of SFN. It should be viewed as complementary to a thorough clinical assessment and preferably investigations, which are more objective measures of small fibre injury.

### Skin biopsy

Quantification of intra-epidermal nerve fibre density is the most important advance in SFN diagnostics over the last decade,^5^ ^33^ and is probably the most validated technique to diagnose SFN. A skin punch biopsy 3 mm in diameter can be taken from any location on the body, but is typically taken 10 cm proximal to the lateral malleolus for diagnostic purposes in SFN.^34^ An additional frequently-used biopsy site is the proximal thigh 20 cm below the iliac spine, which in combination with the distal biopsy can help differentiate between a neuropathy and a neuronopathy.^35^ An alternative method to the punch biopsy is to take a sample of tissue via the skin blister technique.^36^ A potential benefit is that no topical anaesthesia is needed, and bleeding is minimal. Although values correlate with those from punch biopsy, additional studies are needed to establish normative values and the skin blister technique's reliability in the diagnosis of SFN.^37^ Skin punch biopsy of the distal leg is very well tolerated with a very low complication rate^12^ and can be performed in almost all patients apart from those with local skin abnormalities.

Small nerve fibre morphometric analysis is performed using bright field immunohistochemistry or indirect immunofluorescence. Bright field immunohistochemistry is the most commonly used technique for routine diagnostics. The skin sample is stained for an antigen called PGP 9.5 (an ubiquitin hydrolase) that is found in all nerve fibres^38^ ^39^ (figure 1). Intra-epidermal nerve fibre density measurements can be established in most neuropathology laboratories as it uses commonly used immunohistochemical techniques. It is important that local normative control counts are compared to international standards before provision of a diagnostic service. It is also possible to send skin samples via courier from external hospitals to central neuropathology laboratories for analysis and there is no reason why this should not be requested by general neurologists. The unmyelinated fibres located in the epidermis/dermis are the terminal nerve endings of either Aδ-fibres or C-fibres.^35^ The standard measure to assess for a SFN is to measure intra-epidermal nerve fibre density, that is, the number of fibres that cross the dermal–epidermal junction per millimetre of epidermal surface.^34^ A decrease in intra-epidermal nerve fibre density with values below the fifth centile relative to age and gender-matched controls are considered diagnostic of SFN,^40^ and decreases in intraepidermal nerve fibre density have been correlated with neuropathy symptoms, and abnormalities on sensory testing.^35^ Intra-epidermal nerve fibre density has been measured in a wide variety of conditions and show consistent results, and in studies where SFN was clinically suspected, this assessment had a sensitivity of 90%, specificity of 95%, positive predictive value of 95% and negative predictive value of 91% for the diagnosis of SFN.^35^ In addition to the quantitative assessment of skin biopsy, there are qualitative assessments of small nerve fibres, such as axonal swellings as a marker of pre-degenerative changes or weaker PGP 9.5 staining, although these are less reliable diagnostically. Skin biopsy with intra-epidermal nerve fibre density measurements is the diagnostic modality of choice for SFN; nerve biopsy is virtually never performed for a pure SFN.

In vivo corneal confocal microscopy shows promise to assess small fibre innervation non-invasively as it can measure and assess corneal innervation.^41^ The cornea is innervated by Aδ-fibres and C-fibres that originate from the ophthalmic division of the trigeminal nerve. It is the most densely innervated part of the human body and offers a unique window to small fibre innervation, as corneal living tissue can be assessed at a cellular level. Corneal nerve fibre bundle density and tortuosity have been assessed most extensively in patients with diabetes mellitus, but also in Fabry's disease, Charcot–Marie–Tooth disease, idiopathic SFN and in non-length dependent neuropathy. Corneal nerve fibre bundle density inversely correlates with severity of neuropathy; therefore, the fewer the nerve fibre bundles the more severe the neuropathy.

### Autonomic nervous system testing

The objective assessment of the autonomic nervous system is not trivial.^1^ If there is suspected autonomic dysfunction on the basis of symptoms and examination, we recommend cardiovascular reflex testing, such as the Ewing protocol,^25^ ^42^ as an initial assessment of the autonomic nervous system, with subsequent referral for a more comprehensive assessment to include tests such as the sympathetic skin response. A further development is that skin biopsies may eventually be used to assess the autonomic nervous system as new techniques have been developed to assess sweat gland and pilomotor muscle innervation, and found to correlate with autonomic dysfunction. However, we need more research before it can be used in routine clinical practice.^1^

### Diagnostic approach to SFN

**Figure 5:**
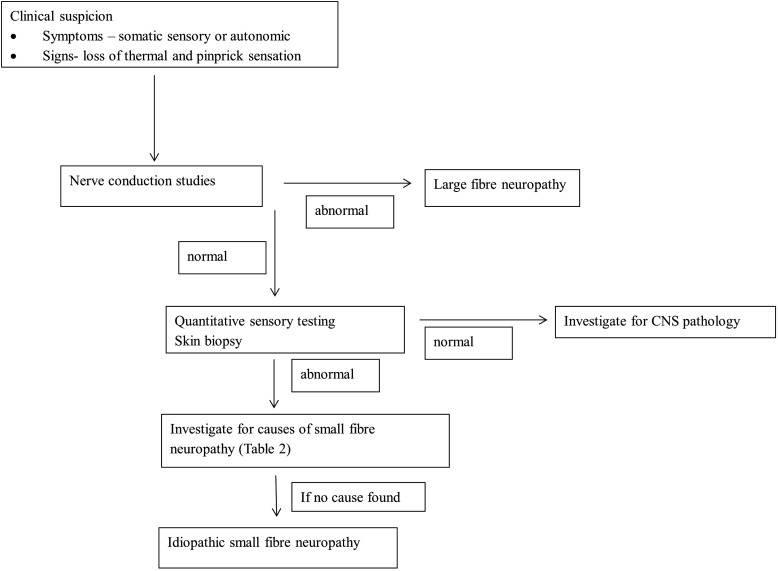
Algorithm for diagnosis of small fibre neuropathy.

In patients where SFN is suspected, the first step is to confirm the diagnosis by following the diagnostic criteria outlined above (figure 5 and clinical vignette). In patients with a pre-existing risk factor such as diabetes mellitus or HIV, it may not be necessary to investigate further, unless there is a clinical suspicion of an exacerbating factor. In patients with no pre-existing risk factor the investigation of an underlying cause should be systematic and appropriate for the patient population. Possible causes of SFN need to be considered and appropriate investigations organised (table 2). If there is no underlying cause, we recommend referral for screening for an *SCN9A* mutation, particularly in patients with an onset of symptoms before the age of 40 and a family history.

**Table 2:** Investigations to determine aetiology of small fibre neuropathy. First line tests are determined by patient population and clinical suspicion.

Blood tests	HBA1_C_, oral glucose tolerance test, urea and electrolytes, thyroid stimulating hormone, HIV serology, hepatitis C serology/viral load, ESR, ACE level, ANA, anti-Ro/La antibodies, rheumatoid factor/anticyclic citrullinated peptide antibodies, antitissue transglutaminase antibody, serum electrophoresis, vitamin B12 levels, leucocyte α-galactosidase A activity (Fabry's disease), lipid profile
Genetic testing	*SCN9A*/*SCN10A* mutations, transthyretin mutations (familial amyloid)
Imaging	If malignancy or sarcoidosis suspected chest X-ray/CT chest with contrast, SAP scan (amyloid)
Tissue biopsy	Abdominal fat biopsy (amyloid), small bowel biopsy (coeliac disease), biopsy of suspicious lesion to confirm malignancy, lip biopsy (Sjögren's syndrome), nerve biopsy is generally not performed unless there is large fibre involvement

ESR, erythrocyte sedimentation rate; SAP, serum amyloid P component.

## Painful channelopathies

There are several rare heritable conditions where small fibres are dysfunctional (leading to severe pain); however, the peripheral small fibres are structurally intact with no evidence of a small fibre neuropathy.

### Inherited erythromelalgia and paroxysmal extreme pain disorder

**Figure 6:**
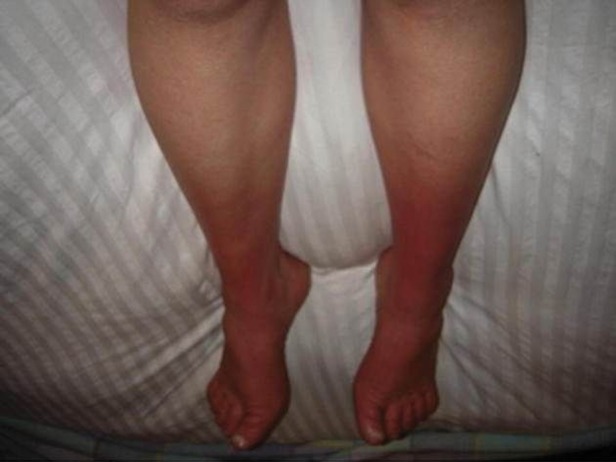
Photograph of the legs of a patient with inherited erythromelalgia, showing erythema to the level of the mid-calf. With increasing ambient temperature, this erythema becomes more extensive. (Reproduced with permission of the International Association for the Study of Pain (IASP) from Segerdahl *et al*).^47^

Inherited erythromelalgia (IE)^43^ and paroxysmal extreme pain disorder (PEPD)^44^ are caused by mutations of the *SCN9A* gene that encodes the sodium channel Na_v_ 1.7.^3^ IE is characterised by episodes of symmetrical burning pain of the feet or legs, often accompanied by reddening of the extremities (figure 6). IE needs to be differentiated from erythromelalgia due to secondary causes such as blood dyscrasia. Typically IE has a family history although there are sporadic cases^45^ Its usual onset is in the first two decades of life, whilst secondary causes have a later onset; there are exceptions with reported cases of IE onset in the sixth decade of life, particularly in the context of mutations with a subtle effect on ion channel function.^46^ The attacks are often triggered by mild warming stimuli (eg, wearing socks or exercise), and many patients report that cooling of the affected limb can ameliorate the pain (a finding which correlates with changes in perfusion of the ‘pain matrix’ assessed using functional brain imaging).^47^ Dominant gain of function mutations in Na_v_1.7 cause IE. The pathophysiological effects of these mutations include: lowered threshold for activation thus allowing the channel to be activated by smaller than normal depolarisations, slowed deactivation thus the channel is kept open for longer once activated and enhanced response to subthreshold stimuli.^48^ The end result is dorsal root ganglion cell hyperexcitability.

PEPD is characterised by paroxysmal attacks of pain involving the lower body (eg, rectum), eyes, and jaw often accompanied by flushing of the affected site and other autonomic disturbances.^44^ The attacks may be precipitated by defecation, crying or yawning or they can occur spontaneously. In contrast to IE, the *SCN9A* mutations associated with PEPD alter Na_v_1.7 by impairing inactivation of the channel leading to an enhanced persistent current.^44^ A key unanswered question is why the Na_v_1.7 mutations associated with IE and PEPD cause such different phenotypic expression particularly in terms of the distal predominance of symptoms in the former and proximal in the latter.

### Familial episodic pain syndrome

Familial episodic pain syndrome was recently described in a Colombian family and is characterised by episodes of debilitating upper body pain that begins in infancy and triggered by fasting or fatigue.^4^ Other contributing factors include illness, cold temperature and physical exertion. The syndrome is caused by an autosomal-dominant mutation in the gene for TRPA1. TRPA1 is a non-selective cation channel selectively expressed by a sub-population of nociceptors, and can be activated both by endogenous ligands and extreme cold and environmental irritants.^49^ The mutation alters the biophysical properties of the TRPA1 channel causing an increase in current flow through the activated channel.

## Management

The key to the management of SFN is to identify any potential treatable cause as there are no treatments that can prevent or reverse SFN. For example, appropriate control of diabetes mellitus can halt its progression and there have been isolated reports of regression of neuropathy after pancreatic transplants.^50^ Immune modulatory therapy, such as intravenous gamma globulins, can reduce the pain associated with celiac disease^51^ and Sjögren's syndrome^52^; however, more research is needed to confirm efficacy of immunomodulatory therapy. In Fabry's disease, the replacement of α-galactosidase A reduces neuropathic pain and can restore warm and cold thresholds and the sweating reflex.^53^ Amyloid is a potentially fatal multi-system disorder; there are treatments for both familial (liver transplant) and acquired amyloid (chemotherapy) that can halt the progression and in some cases improve peripheral neuropathy.^18^ Unfortunately, in certain conditions such as HIV, the progression of SFN is not altered by successful suppression of the HIV.^54^ It is also important to exercise caution in prescribing potentially neurotoxic agents, such as certain antibiotics (table 1) in those patients with an underlying SFN.

Unfortunately, we have no neuroprotective therapies for SFN. A number of trials have been undertaken to regenerate nerve fibres (particularly in diabetic neuropathy) through the administration of neuroprotective factors, such as nerve growth factor.^55^ These trials showed lack of efficacy and in some cases dose-limiting side effects. Therefore, once the underlying aetiology has been successfully managed the treatment of complications takes priority. Pain and autonomic dysfunction are the major problematic complications of SFN. Both are difficult to treat, and current treatments are far from satisfactory.

There are no guidelines specifically for pain associated with SFN. Recently, consensus guidelines for neuropathic pain have been adapted for treatment of pain in SFN.^2^ Pragmatic consensus guidelines include those of the UK's National Institute of Health and Care Excellence and the Map of Medicine.^56^ This usually involves first-line treatment with gabapentinoids, gabapentin or pregabalin (that bind to α_2_δ_1_ and alter trafficking of voltage-gated calcium channels), tricyclic antidepressant (unlicensed indication) or serotonin-norepinephrine reuptake inhibitor (licensed for painful diabetic neuropathy). Drugs must be introduced in a step-wise manner and titrated for efficacy and side effects.^13^ A common problem is insufficient dose titration. There is some evidence for combining drug classes to treat neuropathic pain.^57^ ^58^ There is now increasing use of topical therapies such as 5% lidocaine plasters, which are helpful if the pain is focal (eg, mainly on the soles of the feet). Treatment with high-dose (8%) capsaicin cream causes desensitisation of cutaneous nerve fibres, and although early trials showed some efficacy in painful HIV neuropathy^59^ this has not been replicated in all trials^60^ and needs to be given in the setting of a specialist clinic. If pain is severe and first-line treatment unsatisfactory, referral to a multidisciplinary pain clinic is essential to ensure holistic care for the patient. Adjuncts such as psychology assessment and management strategies can be extremely helpful. Our policy is to assess those patients in whom pain is not adequately controlled in a joint clinic with pain physicians with access to psychology support. Treating autonomic dysfunction is difficult, but can respond to specific interventions.^61^ It is difficult to be definitive as to duration of follow-up for patients with SFN. There needs to be a thorough investigation for underlying cause and we would normally assess at least up to one year in order to check for progression. Patients need long-term follow-up for pain management, which a pain clinic can provide.

Treatment of painful channelopathies such as erythromelalgia is also extremely challenging.

PEPD responds to carbamazepine; however, carbamazepine efficacy in IE is less predictable. Mexiletine may also be used. The response to therapy depends on the exact mutation; both carbamazepine and mexiletine are effective in terms of pharmacology assessed in vitro and clinically. Prediction of efficacy may be improved in future using structural modelling.^62^ There is a considerable drive currently to develop more specific blockers of Na_v_ 1.7 and a number of agents are currently in clinical trials.

## Clinical vignette

A 44-year-old woman presented with a 6-month history of severe burning pain in the soles of both feet. This progressed to the level of the knees and also affected the hands. She described several episodes of her heart racing and recently had developed altered bowel habit with alternating constipation and diarrhoea. Her symptoms were disabling such that she patient had to stop work as a management executive. On examination, she had a resting tachycardia but there was no postural drop, and no organomegaly or lymphadenopathy. Tone, power and coordination were normal, and all deep tendon reflexes were preserved. On sensory examination, pinprick and temperature sensibility were reduced to the knees bilaterally; light touch, proprioception and vibration sense were normal.

Nerve conduction studies were normal; however, a skin biopsy taken from the leg 10 cm above the lateral malleolus showed a markedly reduced intra-epidermal nerve fibre density of 0.2/mm, confirming the clinical diagnosis of SFN.

The patient was found to have an IgG κ paraprotein 10 g/L. Investigations for AL-amyloid light-chain were therefore initiated. Skeletal survey, urinary Bence–Jones protein and bone marrow examination were normal/negative. A serum amyloid P scan (associated with lower morbidity compared with nerve biopsy) showed amyloid deposition in the liver and spleen. Abdominal fat biopsy was negative for amyloid deposition; however, a subsequent rectal biopsy confirmed amyloid deposition. She started treatment with Lenalidomide, following which the level of serum-free light chains dropped; 1 year later, the pain was much improved with clinical evidence of improvement in the neuropathy and she returned to work.

This case illustrates the point that amyloid neuropathy can present as a pure SFN and in the context of normal nerve conduction studies, skin biopsy was helped in confirming the diagnosis. SAP scans often show organ involvement not thought to be involved clinically and are most sensitive to amyloid deposition in large visceral organs.^63^ Chemotherapy regimens are based on the treatment of myeloma and have improved the prognosis of AL amyloidosis.

**Key points**

- Small fibre neuropathy is defined as a structural abnormality of small fibres characterised pathologically by degeneration of the distal terminations of small fibre nerve endings.
- Gain of function mutations of the genes *SCN9A* or *SCN10A* that encode the voltage-gated sodium channels Na_v_ 1.7 and Na_v_ 1.8 are associated with previously unexplained small fibre neuropathy.
- The diagnosis of small fibre neuropathy is best made through the combination of clinical signs of small fibre dysfunction and assessment of intra-epidermal nerve fibre density. Corneal confocal microscopy is a new and promising non-invasive means of assessing structural integrity of small fibres.
- The key to management of small fibre neuropathy is to identify any potential treatable causes and to focus on pain management where possible in a multidisciplinary setting.
